# Host miRNAs-microbiota interactions in gastric cancer

**DOI:** 10.1186/s12967-022-03264-3

**Published:** 2022-01-29

**Authors:** Yan Yang, Yingying Huang, Wu Lin, Jin Liu, Xiangliu Chen, Chuanzhi Chen, Xiongfei Yu, Lisong Teng

**Affiliations:** 1 Department of Surgical Oncology, The First Affiliated Hospital, School of Medicine, Zhejiang University, Hangzhou, 310003 China; 2 Clinical Research Center, The First Affiliated Hospital, School of Medicine, Zhejiang University, Hangzhou, 310003 China

**Keywords:** Gastric cancer, miRNAs, Microbiota, Metabolic interactions, Microenvironment

## Abstract

It is widely acknowledged that gastric cancer seriously affects the quality of life and survival of patients. The correlation between the microbiota and gastric cancer has attracted extensive attention in recent years, nonetheless the specific mechanism of its impact on gastric cancer remain largely unclear. Recent studies have shown that in addition to its role in the host’s inflammatory and immune response, the microbiota can also affect the occurrence and development of gastric cancer by affecting the expression of miRNAs. This paper brings together all currently available data on miRNAs, microbiota and gastric cancer, and preliminarily describes the relationship among them.

## Background

Gastric cancer (GC) is the fifth most common malignant tumor globally, after lung, breast, colorectal and prostate cancers. Even though its incidence rate has declined in recent years, it is still the third leading cause of cancer-related death worldwide [1, 2]. In China, GC ranks third in the incidence and tumor-associated death in the national population-based cancer registry [3]. This finding can be attributed to the fact that most patients with gastric cancer are already at an advanced stage at diagnosis, which is correlated to the high incidence of postoperative local recurrence and distant metastasis and the poor treatment outcomes [4].

The human gastrointestinal microbiota constitutes a complex micro-ecosystem involved in metabolism, promoting the development of the immune system and inhibiting the colonization of pathogens. With the development of sequencing technology, more strains have been found to colonize the stomach. Aviles et al. found decreased numbers of *Porphyromonas*, *Neisseria* and *Streptococcus* and increased *Lactic acid bacteria*, *Pseudomonas aeruginosa* and *Trichospirillum* in gastric cancer by comparing the differences in gastric microbiota of patients with non-atrophic gastritis, intestinal metaplasia and intestinal-type gastric cancer. This finding suggests that these bacteria may play a role in the development of gastric cancer from gastritis [5]. Recent studies on the microbiota in gastric cancer and paracancerous tissues have shown that the mucosa-associated microbiota in tumor tissues and non-tumor tissues have different established microecosystems in terms of composition, structure, interaction network and function. In this regard, it has been shown that *Proteobacteria* are the main phylum, followed by *Firmicutes*, *Bacteroides*, *Actinomycetes*, *Acidobacteria* and *Fusobacteria* in tumor tissues, and acid-producing bacteria are more abundant in non-tumor tissues [6]. Another team analyzed the microbiota of gastric cancer patients’ tumor tissues, adjacent tissues and normal tissues, found significant differences in the microbiota among the three groups. Furthermore, another study demonstrated a higher abundance of *Spiricobacter*, *Halomonas* and *Shevchella* in adjacent tissues, while *Streptococcus*, *Selenomonas*, *Fusobacterium*, *Propionibacter* and *Corynebacterium* were more abundant in tumor tissues [7].

**Fig. 1:**
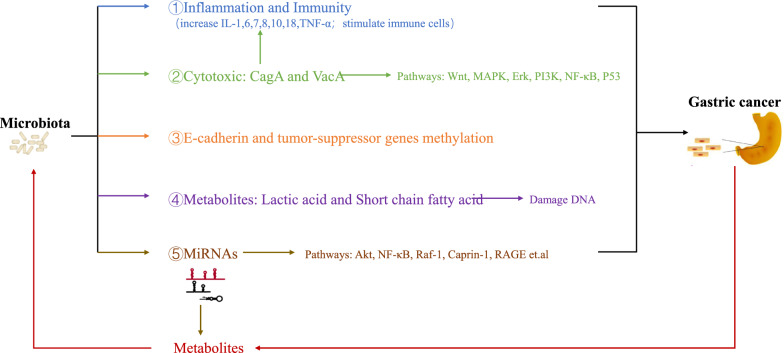
Host miRNAs-microbiota interactions in gastric cancer. Microbiota can affect the development of gastric cancer by five ways:①, ②Microbiota directly or through cytotoxic affect host inflammation and immune response;③Microbiota induced methylation of E-cadherin and tumor-suppressor genes;④Microbiota damage host DNA through metabolites;⑤Microbiota can also affect the expression of host miRNAs, which can affect the occurrence and development of gastric cancer. Tumor metabolites can affect microbial colonization, while miRNAs can also affect host metabolism and indirectly affect microbial colonization

An increasing body of evidence suggests that the microbiota can affect the occurrence and development of tumors in various ways, including chronic inflammation and immune regulation [8–16]. In recent years, it has been shown that microRNAs (miRNAs) can influence the survival and composition of gut bacteria [17–19]. Based on the existing literature, we hypothesized interactions between the microbiota and the host miRNA affect the occurrence and development of tumors. In this review, we present our current understanding of microbiota-miRNAs interactions in gastric cancer (Fig. 1). After summarizing evidence pointing to their core role, we look forward to the future directions of this rapidly developing field.

## Microbiota and gastric cancer

### Microbial imbalances affect the occurrence and development of gastric cancer

Many studies have confirmed significant differences in microbial composition between gastric cancer patients and healthy controls, indicating that an imbalanced gastric microbiome is an important cause of gastric cancer [6, 7]. Gastric cancer is driven by inflammation-related factors as many studies have shown that the microbiota can promote the development of gastric cancer by promoting inflammation and regulating immunity.

Previous studies that explored the effect of microbiota on gastric cancer mainly focused on *Helicobacter pylori* (Hp)*.* Marshall and Warren identified Hp from a gastric biopsy culture in 1984 [20]. Nowadays, it is widely recognized that Hp infection is a high-risk factor for gastric cancer [21]. In this regard, Hp infection can increase secretion of inflammatory cytokines such as interferon-c, TNF-a, IL-1, IL-1B, IL-6, IL-7, IL-8, IL-10 and IL-18, which stimulate a variety of immune cells, including lymphocytes, peripheral monocytes, eosinophils, macrophages, neutrophils, mast cells and dendritic cells [8]. Hp infection also can cause methylations on E-cadherin and tumor-suppressor genes [22]. The cytotoxin-associated gene A (CagA) and vacuolating cytotoxin gene (VacA) are the main virulence factors of Hp. Both CagA and VacA can cause inflammatory reactions and affect the occurrence and development of tumors. CagA can promote the Wnt, MAPK, Erk and NF-κB pathways and inhibit the activity of p53 [23–25]. VacA can induce autophagy by upregulating MAPK and ERK signaling, directly activating mitochondria and vascular endothelial growth factors. VacA has also been reported to act on the Wnt pathway and pI3k/Akt signaling pathways [26–30]. Some studies have documented interactions between CagA and VacA, which exhibit antagonistic effects on the NFAT pathway and cell morphology. Importantly, CagA can also inhibit the apoptosis of VacA [31].

In recent years, with the development of sequencing technology, an increasing number of microbes have been discovered in the stomach, such as *Firmicutes*, *Proteobacteria*, *Bacteroidetes*, *Fusobacteria* and *Actinobacteria *[32]. Nonetheless, few studies have documented the effect of other microbiota on gastric cancer. Indeed, other bacteria can also affect the occurrence and development of gastric cancer through regulatory pathways, immune cells and their metabolites. For example, *Stenoprophomonas* and *Selenomonas* are increased in gastric cancer and positively correlated with BDCA2 + pDCs and Foxp3 + Tregs, while *Comannas* and *Gailla* are negatively correlated with BDCA2 + pDCs and Foxp3 + Tregs [33]. Further studies have confirmed that the abundance of *Fusobacterium* in gastric cancer is associated with a poor prognosis [34–36]. *Fusobacterium* infection has also been associated with tumor-infiltrating lymphocytes and p53 expression [34]. However, the specific mechanisms on gastric cancer progression remain understudied. Interestingly, the levels of *P.copri* and *P. acnes* are reportedly increased in gastric cancer patients, affecting the occurrence and development of gastric cancer through the NKG2D system and IL-15 [37]. In contrast, some studies have shown that *P.copri* levels were decreased in gastric cancer [7]. N-nitroso compounds (NOCs) and lactic acid have been widely reported as the main microbial metabolites associated with gastric cancer. NOCs are produced by many bacteria such as *Clostridium*, *Veillonella*, *Staphylococcus*, and *Lactobacillus* that are increased in gastric cancer [38]. It is also well established that NOCs are associated with gastric cancer. ROS produced by lactic acid bacteria can damage DNA, promote tumor growth and metastasis, and inhibit tumor apoptosis by promoting NOCs production [33, 39]. Many studies have reported that the abundance of *Lactobacillus* in patients with gastric cancer is increased, and their lactic acid metabolites can affect the occurrence and development of gastric cancer [40, 41].

### Factors affecting microbial colonization

Over the years, an increasing number of studies have emphasized the importance of the balance in the microbiota that colonizes the human body. Microbial imbalance is also an important factor responsible for cancer, described in detail in the previous paragraph. Importantly, microbial colonization of gastric cancer can be affected by the microenvironment (microbial interaction, surgical operation, stage or type of GC and so on) [42–46].

Studies have shown that Hp infection can change the microbial composition of gastric cancer patients [43]. A decreased microbial diversity has been reported in the stomach of HP-positive patients; the main microorganisms were *Proteus*, followed by *Firmicutes*, *Bacteroidetes* and *Actinomycetes *[47]. Similarly, Anderson et al. found 33 phylotypes in the stomach of HP-positive gastric cancer patients and more than 200 phylotypes in healthy controls [48]. Importantly, genes related to nucleotide transport metabolism and amino acid transport metabolism are significantly enriched in patients’tumor microbiota, and gastric acid secretion increased significantly in the Hp-positive gastric cancer patients [7]. Moreover, HP may lead to a gastric inflammatory environment through CagA and VacA virulence factors, thus affecting the growth of other microorganisms [49]. Leung et al. analyzed the characteristics of individuals with gastric cancer with different histological stages and the gastric microbiota after Hp eradication. Importantly, HP eradication increased the bacterial diversity, and the relative abundance of other bacteria returned to a level similar to healthy controls [50]. Similarly, the gastric microbiota and their metabolites can influence the ability of Hp to colonize the stomach and modulate its pathogenicity and carcinogenic potential [44].

Elaine et al. showed that the metabolism and microbial composition of gastric cancer patients were significantly altered after Roux-en-Y gastric bypass (RYGB) surgery [45]. Consistently, Vaidota et al. showed that gastric cancer surgery could affect the composition of gastric microbiota [46]. Liu et al. found that the gastric microenvironment determined the composition and diversity of gastric microbiota rather than the stage or type of GC. The above studies substantiated that the microbiota could affect the occurrence and development of tumors; however, it should be borne in mind that the tumor microenvironment can also regulate the microbial composition.

## MiRNAs and gastric cancer

MiRNAs belong to non-coding RNAs that function by regulating downstream target genes. The imbalance of miRNAs is closely related to the occurrence and development of gastric cancer. Besides, the stability of miRNAs enhances their potential as tumor biomarkers. The role of miRNAs during the pathogenesis of gastric cancer and their potential application as biomarkers and therapeutic targets for gastric cancer has attracted much interest.

### Clinical application of miRNAs

It has been established that miRNAs can be released from tumor tissue to the serum, plasma, urine, tears, amniotic fluid and gastric juice through exosome particles. Studies have confirmed that circulating miRNAs in the plasma/serum of GC patients are consistent with miRNA expression levels in tissues. Accordingly, they can be used as biomarkers for early diagnosis and recurrence evaluation of gastric cancer [51]. Table 1 lists some circulating miRNAs associated with gastric cancer based on serum/plasma, considered as biomarkers of gastric cancer. Previous studies have shown significant differences in circulating miRNAs between patients with gastric cancer and normal controls.

**Table 1:** MiRNAs as biomarkers for gastric cancer

MiRNAs	Samples	Sensitivity/specificity	Type of biomarker	Study
miR-199a-3p	Plasma	0.76/0.74	Diagnostic	[56]
miR-451	Plasma	0.96/1	Diagnostic	[57]
miR-486	Plasma	0.86./0.97	Diagnostic	[57]
miR-21	Serum	0.88/0.79	Diagnostic, prognostic	[58]
miR-200c	Blood	0.65/1	Diagnostic, prognostic	[59]
miR-221,miR-744,miR-376c	Serum	0.82/0.58	Diagnostic, prognostic	[60]
miR-940	Plasma	0.81/0.98	Diagnostic	[61]
miR-551b-5p	Serum	0.77/0.8	Diagnostic	[62]
miR-19b,miR-106a	Circulating exosomes	0.95/0.9	Diagnostic, prognostic	[63]
miR-146,miR-375,let-7,miR-19,miR-21	Plasma	N/S	Diagnostic	[64]

Recent studies have shown that the serum levels of EV-miR-215-5p in gastric cancer patients were significantly higher than in the healthy subject group, especially in patients with early gastric cancer recurrence. Importantly, serum EV-miR-215-5p levels were significantly correlated with the depth of invasion, TNM stage and lymph node metastasis. Accordingly, serum EV-miR-215-5p has huge prospects as a new biomarker for predicting early gastric cancer recurrence and prognosis of gastric cancer [52].

Wang et al. showed that miR-18a-5p could be a potential therapeutic target for gastric adenocarcinoma [53]. The combination of miR-4257, miR-6785-5p, miR-187-5p and miR-5739 provided a useful and high-precision non-invasive diagnostic means for the detection of early gastric cancer in a large sample size study (n = 1417) [54]. Importantly, Wang et al. showed that miR-214 could be used as a target in patients exhibiting chemotherapy resistance; the injection of exo-anti-214 could reverse the resistance of gastric cancer patients to cisplatin [55]. Overall, the above studies demonstrate that miRNAs have great potential for clinical application when used as biomarkers for diagnosis, prognosis and treatment.

### MiRNAs affect the development of gastric cancer

MiRNAs can be used as biomarkers of gastric cancer since they can affect the development of gastric cancer in many ways. It has been found that different miRNAs have different effects on gastric cancer; they can promote or inhibit gastric cancer. miR-96-5p is reportedly overexpressed in gastric cancer cell lines and plasma samples of gastric cancer patients and can promote the proliferation of gastric cancer cells by directly targeting FOXO365. Importantly, it has been shown that miR-93-5p overexpression in gastric cancer cells upregulates the downstream genes CDX2, FoxM1 and CTGF of the Hippo pathway [66]. However, another study has shown that miR-146b-5p is negatively correlated with lymph node metastasis and distant metastasis of gastric cancer and inhibits the malignant development of gastric cancer by targeting TRAF6 [67]. Low expression of miR-339-5p has also been documented to be closely related to metastasis and prognosis of gastric cancer, and miR-339-5p can inhibit the malignant development of GC by negatively regulating ALKBH1 [68]. Table 2 lists the latest studies on the promotion or inhibition of miRNAs on gastric cancer.

**Table 2:** MiRNAs in cell proliferation, cell cycle, and apoptosis

MiRNAs	Samples	Cell functions	Target	Study
miR-28-5p	GC tissues and adjacent normal tissues; GC cell lines:BGC823,SGC7901	Inhibition of proliferation and migration of gastric cancer cells	AKT	[69]
miR-7	GC samples and matched non-GC samples; Cell lines: normal cell line(GES-1), GC cell lines(HEK-293 T,SGC-7901,BGC-823,HGC-27,MKN-28,MKN-40);Serum	Inhibition of GC metastasis; Suppress tumor development and angiogenesis of GC cells	NF-κB; Raf-1	[70, 71]
miR-181a	GC tissues and adjacent normal tissues; GC cell lines:MKN45,SGC-7901,MGC803,BGC-823,HEK-293 T;Normal cell line:GES-1	Inhibit apoptosis and promote the proliferation, invasion and metastasis of gastric cancer cells	Caprin-1	[72]
miR-1915	20 pairs of H. pylori-positive and negative gastritis tissues, 30 pairs of GC tissues and adjacent normal tissues; GC cell lines: SGC-7901, MKN-45;Normal cell line:GES-1	Inhibits proliferation, invasion, and migration of Helicobacter pylori-Infected gastric cancer cells	RAGE	[73]
miR-1299	GC tissues and adjacent normal tissues; GC cell lines: AGS and SGC7901	Inhibits cell proliferation, migration and invasion, and promotes cell apoptosis	ARF6	[74]
miR-133a-5p	GC tissues and adjacent normal tissues; GC cell lines: MGC-803, BGC-823, HGC-27, SGC-7901; Normal cell line:GES-1	Inhibits cell growth and metastasis, and promotes apoptosis	TCF4	[75]
miR-653-5p	80 pairs of GC tissues and adjacent normal tissues; GC cell lines: MGC803, BGC823, SGC7901, MKN45, AGS; Normal cell line:GES-1	Promotes gastric cancer proliferation and metastasis	SOCS6-STAT3	[76]
miR-135b	de-identified 146 GC tissuesand 28 pairs of GC tissues; Blood samples from 23 GC patients and 27 health controls; GC cell lines: MKN28 and BGC823; Normal cell line:GES-1	Promotes cell proliferation, migration and invasion	CAMK2D	[77]
miR-30a-3p	GC cell lines: SGC7901, MGC803, and MGC823; Normal cell line:GES-1	Inhibits the development of gastric cancer	APBB2	[78]
miR-1301-3p	GC and normal tissues from sixty patients; GC cell lines:MGC-803 and SGC-7901	Promotes gastric cancer cell proliferation, cell cycle progression and tumorigenesis	SIRT1	[79]

### Host miRNAs affect cancer metabolism

Changes in host miRNAs can change the metabolism of tumors. Cao et al. showed that the circlmo7-miR-30a-3p-wnt2 axis could provide energy for the growth of GC cells through glutamine metabolism [80]. Enhanced miR-4646-5p can stabilize HIF1A by targeting PHD3 and regulating the expression of ABHD16a and miR-4646-5p itself via a positive feedback mechanism. As a novel phosphatidylserine-specific lipase, ABHD16a participates in lipid metabolism and accumulates lysophosphatidylserine [81]. Other studies have also corroborated that miR-34a has a regulatory effect on lactic acid [82].

## Microbiota affect miRNAs expression

An increasing body of evidence suggests that Hp infection can affect host miRNAs expression. For instance, Chang et al. showed that in Hp-positive gastric cancer patients, miR99b-3p, miR-564, and miR-638 were significantly increased, while miR-204-5p, miR-338-5p, miR-375, and miR-548c-3p significantly decreased, compared with Hp-negative patients, [83]. Moreover, miR-18a-3p and miR-4286 have been reported to be induced by Hp infection, while miR-204 was decreased. Previous studies also showed that above miRNAs induced the NF-κB pathway, promoting the growth of gastric cancer [84, 85]. In addition, the expression of many miRNAs is affected by Hp infection, such as miR-146a, miR-1289, miR-223-3p, miR-370, miR-22, miR-133 and so on [86–89].

Interestingly, after EBV enters the human body, it can cause host genome methylation, abnormal gene expression, disrupt cell signaling pathways, and generate a tumor microenvironment in infected gastric epithelial cells, leading to the occurrence and development of gastric cancer [90]. In addition, latent EBV gene products, such as EBERS, BARF-0, EBNA-1 and LMP2A, are reportedly involved in downregulating the miR-200 family, resulting in decreased expression of E-cadherin or inducing NF-κB/miR-146a/Smad4 pathway in gastric cancer cells. They can also upregulate miR-155-5p expression by NF-κB pathway and inhibit the activation of Smad2 and p-Smad2 [91–93].

## Perspective

MiRNAs play a key role in the interaction between microbiota and host. The microbiota can affect the occurrence and development of cancer directly or indirectly. Studies have shown that HP infection can increase inflammatory factors such as IL-1, 6 and 7, thus affecting the body's immunity [8]. HP infection can also increase the methylation of E-cad and tumor suppressor genes [22]. Meanwhile, it can also promote Wnt, MAPK, ERK, NF-κB, PI3K pathway and inhibit the p53 pathway by secreting CagA and VacA [21, 25, 87]. At present, a large number of studies have shown that *Fusobacteria*, *Actinobacteria*, *Proteobacteria* and *Bacteroidetes* are related to gastric cancer, and some strains are significantly related to tumor microenvironment immune factors [32, 34]. Lactic acid and NOCs produced by many strains can also promote tumor development by destroying DNA [33]. Nonetheless, the mechanisms underlying the above findings remain understudied, warranting further studies.

Previous studies have found that miRNAs can be used as potential clinical biomarkers. For example, a significant difference in the expression levels of miR199a-3p, miR-451 and other miRNAs was found between cancer patients and normal controls [56, 57] A significant correlation has been reported between patient prognosis and miRNAs miR-21 and miR-200c, which indicates that miRNAs can be used as prognostic markers [58, 59]. MiRNAs can not only be used as biomarkers of gastric cancer, but also affect Akt and Akt, NF-κB, RAGE, ARF6, TCF4, STAT3 and other signaling pathways promote or inhibit the occurrence and development of gastric cancer [69, 70, 73–76]. In the meantime, miRNAs can also alter lipid metabolism, energy metabolism and amino acid metabolism, thus indirectly affecting the occurrence and development of cancer [80–82].

Some studies have also shown that the microbiota can affect the expression of host miRNAs, and miRNAs can conversely affect microbial colonization by changing the host-related metabolism [89]. However, the specific mechanism of the interaction between host miRNA and colonized microbiota in gastric cancer remains largely unexplored. In future research, an interdisciplinary endeavor is necessary to fill the knowledge gap on miRNA-mediated host-microbiome communication and provide key information for developing new treatment strategies to decrease gastric cancer-associated mortality rates.

## Data Availability

Not applicable.
